# A Probabilistic Model for Real-Time Semantic Prediction of Human Motion Intentions from RGBD-Data

**DOI:** 10.3390/s21124141

**Published:** 2021-06-16

**Authors:** Wouter Houtman, Gosse Bijlenga, Elena Torta, René van de Molengraft

**Affiliations:** Control Systems Technology Group, Department of Mechanical Engineering, Eindhoven University of Technology, P.O. Box 513, 5600 MB Eindhoven, The Netherlands; g.bijlenga@newcircletechnologies.nl (G.B.); e.torta@tue.nl (E.T.); m.j.g.v.d.molengraft@tue.nl (R.v.d.M.)

**Keywords:** human intention estimation, probabilistic reasoning, indoor navigation, semantic reasoning

## Abstract

For robots to execute their navigation tasks both fast and safely in the presence of humans, it is necessary to make predictions about the route those humans intend to follow. Within this work, a model-based method is proposed that relates human motion behavior perceived from RGBD input to the constraints imposed by the environment by considering typical human routing alternatives. Multiple hypotheses about routing options of a human towards local semantic goal locations are created and validated, including explicit collision avoidance routes. It is demonstrated, with real-time, real-life experiments, that a coarse discretization based on the semantics of the environment suffices to make a proper distinction between a person going, for example, to the left or the right on an intersection. As such, a scalable and explainable solution is presented, which is suitable for incorporation within navigation algorithms.

## 1. Introduction

Current trends in robotics show a transition to environments where robots in general, and Automated Guided Vehicles (AGVs) in particular, share the same space and collaborate with humans. Examples of such robots are given by the SPENCER-project [1], the ROPOD-project [2] and the ILIAD-project [3], which target, respectively, airports’ guiding assistance, logistical tasks within hospitals and logistic services in warehouses. Safety is an important concern for such systems. To address the safety of a robot’s navigation trajectories in the presence of humans, it is needed to estimate where a person is moving to and take the estimated movement’s direction into account in its own navigation behavior. For example, to prevent collisions, a robot could move to the right when a person is going to pass on its left side or slow down to give priority to a person when going to pass a crossing. By taking a human’s intentions into account, collisions between robots and persons can be prevented while the robots keep on moving efficiently. Most commonly, to guarantee safety, systems are either backed up by a human operator or show conservative behavior by either waiting or driving too slowly. A better understanding of the environment dynamics is required in real-time to prevent dangerous situations [4]. When a robot can predict the walking intention of a human, it could speed up its navigation progress by, for example, continuing to move even if a person is close by but predicted not to intersect its trajectory. A human walking pattern prediction model can help to reduce conservatism in navigation [5]. Following this line of reasoning, the research reported in this paper presents a model that can estimate the walking intention of a human from RGBD-data within (indoor) spaces for which a map with structural semantic elements (e.g., walls and doors) is known. The effectiveness of the model is experimentally validated by means of real-life, real-time experiments. The model is developed by taking into account the following requirements: (1) scalability and applicability to different configurations of the environment, (2) simultaneous evaluation of different and plausible routing alternatives such that navigation algorithms can consider the difference in likelihood of the alternatives, (3) real-time execution, (4) adjustability of the prediction horizon to relate the horizon to the timescale of the navigational task at hand and (5) explainability in the sense that the proposed approach expresses an explicit relationship between, on one hand, measurements andestimations of human motion and postures, and, on the other hand, to elements of the map. This explicit relationship provides predictions at both geometric and semantic levels.

The remainder of this paper is organized as follows: Section 2 presents an overview of literature relevant to the approach presented in the paper. Section 3 presents a methodology to define (a) semantic maps with associated hypotheses on a human’s movements directions and (b) algorithms to compute predictions about the expected human’s movements directions based on the semantic map and the set of hypotheses. Section 4 validates the method with real-time and real-life experiments. Finally, Section 5 concludes the paper and provides suggestions for future work.

## 2. Related Work

This section discusses the work related to human intention prediction in contexts involving robots. First, a discrimination is made based on the methods used by the approaches: data-based and model-based. Then, we report on how semantics have been used to enhance human motion prediction by model-based algorithms. Different discretizations of the space are discussed as well as methods to determine hypotheses on a human’s movements directions. The final paragraph summarizes the contributions of this work.

When robots predict the motion intention of a human with the purpose to adapt their (navigation) behavior, we should consider that the motion of the robot will have an influence on the motion of the human. Considering this situation as a multi-agent problem requires complex approaches, which are difficult to scale and implement in practice, as [5] claims. To reduce this complexity, in the present work, we decided to focus only on the estimation of the navigation intention of humans.

Two general approaches to prediction exist, namely prediction based on explainable models as defined in the requirements and prediction based on training end-to-end algorithms from data acquired offline.

For data-based prediction methods, typical trajectories within a given map are first collected over time. Those then serve to train models that will predict the trajectory of a human based on online observations. Typically, a dense discretization of the space is applied, see, e.g., [6,7,8,9]. Within the works of [10,11], the discretization issue is addressed by learning a topological map, which summarizes a set of observed (person) trajectories. As indicated by the authors of the latter work, the topological map is missing the semantic meaning as the nodes are defined in Cartesian space, rather than relative to semantic elements of the map. This makes it difficult to transfer the learned models to other areas with similar semantic configurations which, we argue, can hamper scalability.

Model-based prediction methods predict the navigation intentions of humans based on explicitly modeling the relation between online measurements, such as velocity and heading direction, and the expected outcome (e.g., see [12,13]). Sometimes, such models take into account the interaction between people and robots, such as the work proposed in [14,15,16,17,18,19]. Future states of the environment are typically modeled as a pose of the person of interest or an occupancy grid of the map where certain cells are expected to be occupied or not [5]. These approaches do not account for the fact that uncertainty about the human intention can give rise to multiple hypotheses. For example, at crossings there are several distinct paths a human could take. In the present work, we take a different approach than what was discussed in [12,13,14,15,16,17,18,19], as we propose a coarse discretization of the environment based on its semantics. As a result, our model does not predict a single path of a human but, instead, it predicts to which semantic location a person is directed to by simultaneously evaluating probable alternatives. In this sense, we argue that if a robot can determine with high accuracy if a person is likely to pass on its left, right or if it is likely to collide, it has enough information to plan its navigation reaction accordingly, guaranteeing safety. This idea of taking the semantics of the environment into account is in line with the suggestions of [20,21]. The work of [21] considers automatic goal inference based on the semantics of the environment as an important future research direction. The authors suggest that intelligent systems should have an in-depth semantic scene understanding and claim that context understanding with respect to features of the static environment and its semantics for better trajectory prediction is still a relatively unexplored field. On this line, our work proposes a methodology which uses the semantics of an indoor environment by matching the human capabilities and the affordances of the semantic environmental map. The notion of affordance refers to the action opportunities provided by the environment [22]. In the context of this work, we consider the possible actions of a person as induced by the environment such as going left or right on a T-junction. Considering such a set of alternatives is in line with concepts from the automotive domain where, for example, a discrimination between walking, running and standing behaviors is predicted or where cyclists’ intentions of going left, right or straight based on cues such as a reaching arm pose [23,24] are predicted. In contrast to these works, in an indoor environment these cues are typically not available and instead of learning the static context within our work we explicitly model it. Looking at the robotics domain, the idea of considering the plausible alternatives for human intentions is in line with [8,19,25]. Within the first of these works, hypotheses about occupied areas in the map are created by considering various trajectories. A dense discretization is applied, which does not scale well. In contrast, our work considers a course discretization by applying areas as a mapping concept [26]. The areas are not physical, but serve as abstract conventions to allow humans and robots to indicate particular parts of the spatial domain [27]. In our work, the discretization of the areas is based on the semantics of the environment. For the second work considering a set of plausible alternatives for human intentions, i.e., [25], intentions are estimated for assistive robotic teleoperation by hypothesizing about the objects present on a table as potential targets. Contrary to our work, the final destinations are considered as the set of plausible alternatives. In order to infer human walking destinations, Kostavelis et al. [19] identified target human locations based on frequently visited spots. The validation of these routing alternatives is based on the assumption that when a human walks towards a target location, the shortest path is, subconsciously, selected. In contrast, our work considered (intermediate) areas as regions of interest, and the robot is explicitly modeled as an object of interest as well.

To recursively update the belief over the hypotheses set, a Bayesian approach is typically adopted [28], which requires the observations to be independent. In the context of this work, the progression of a person towards the area corresponding to a hypothesis is chosen as a measure to compute the likelihood of that hypothesis. We do so by comparing the direction component of the estimated human’s velocity vector to the expected direction of movement corresponding to the hypothesis being evaluated. To properly apply a Bayesian approach, this requires the velocity observations to be independent. This is, in general, not the case for robots within the context considered in this paper, as typically the velocity estimate of a person is based on filtered position observations. Furthermore, the Bayesian approach requires a transition model, which, in our context, needs to represent the probability of a person choosing an alternative direction. To take this property into consideration, specific knowledge about human behavior in the targeted environment is required, which is considered out of scope of this work. Therefore, we opted for a maximum likelihood approach for estimating the likelihood of each hypothesis.

In conclusion, the contribution of this work is a methodology that estimates humans walking intention by

1. posing an abstract semantic model for human intentions that encloses all probable walking paths to predefined semantic goals,
2. evaluating the model as human walking hypotheses.

The result is a robust intention estimator as proven by the real-time and, real-life experiments.

## 3. Methodology

**Figure 1:**
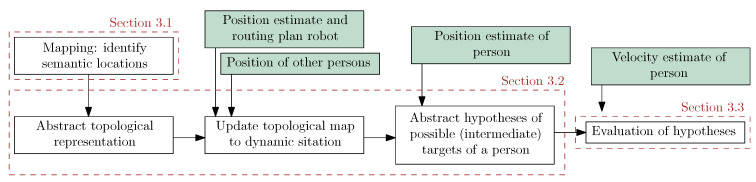
Overview of the steps taken in this work to obtain the most probable hypothesis of the intention of a person in an indoor environment. The green blocks indicate (filtered) observations or information required from other components, which are assumed to be available in the system such as a human detection algorithm.

To predict which possible directions a person is moving to, the model of the human’s capabilities (Section 3.1) is matched with the affordances of a semantic environmental map (Section 3.2). The derived human’s movements hypotheses are evaluated using a maximum likelihood approach (Section 3.3). A graphical visualization of the proposed method is reported in Figure 1.

### 3.1. Definition of the Hypotheses on a Human’s Navigation Goals

We assume that when navigating, humans have an intention towards a specific and semantic goal location that can be extracted from a semantic map. When considering all possible goal locations in a huge building such as a hospital or a warehouse, the amount of hypotheses would be very large and almost intractable. Moreover, not all possible goal locations are relevant for a robot with a navigational task. For example, the structure of a hallway imposes that all goal locations within a specific room lead via the doorway to that specific room. Therefore, in this work, we consider only alternative goal locations that are in the direct neighborhood of the human and robot. The goal locations outside this area of interest are summarized by hypothesizing about the intermediate route towards the destination. In the example of the room in the hospital, the direction to the next hallway is most relevant for a robot that encounters the person moving to that room at a cross-shaped intersection. The other relevant alternatives for a robot passing this intersection are left, right and straight.

For the alternative, where the robot and the person encounter each other, a collision between the human and the system is possible. We have then three alternatives, either the person will avoid the robot passing on the left or on the right or the person will collide with the robot. The discrimination between these alternatives is important for robotic navigation because a robot can act, for example, by moving to the left if the person is passing to the right and the other way around. When people walk, it is common that they stop in a standstill position to, for example, check their phone and do not make any progression towards their desired destination goal. This hypothesis is also taken into account in our framework. Lastly, we acknowledge that people might have another goal compared to those modeled. We represent this explicitly by an Undefined Goal (UG) hypothesis. In summary, as listed in Table 1, given a semantic map of the environment, we consider the following hypotheses to describe every encounter between the robot and a human as:

1. All (semantic) directions that lead to an alternative route such as left, straight and right on a crossing or an object of interest such a person or a locker (one hypothesis for each direction based on the position of the person inducing different directions of movement);
2. The alternatives in the direct neighborhood of the robot, namely;
  (a) Passing of the robot either on the left side or on the right side (two hypotheses leading to different directions of movement);
  (b) The collision with the area required for navigation of the system (single hypothesis with a movement directed towards the robot);
3. A standstill of the person (single hypothesis with zero velocity);
4. Undefined Goal, i.e., not 1–3 (single hypothesis when there is no evidence for the alternative movement directions).

### 3.2. Linking Semantic Maps to the Hypotheses

**Figure 2:**
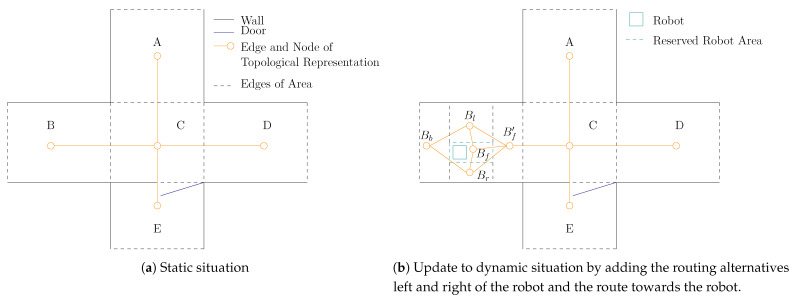
Example of the creation of areas and the topological representation of a map consisting of an intersection C including a doorway and the corridors A, B, D and E connected to this intersection.

As stated earlier, we determine the applicable set of hypotheses from a semantic map, which is assumed to be known a priori. Within this semantic map, the concept of areas is adopted as proposed by [26,27]. In general, areas serve as abstract conventions to allow humans and robots to indicate particular parts of the spatial domain [27]. For the purpose of considering the semantics of the environment to predict the direction of the movements of humans, the division of the map in areas should represent the various routing alternatives. Indicators for the distinction are, for example, corridors, crossings, T-junctions and passages such as doors. In the example of Figure 2a, this leads to the discrimination of junction *C*, which has the routing alternatives to corridors *A*, *B* and *D*. For the alternative to corridor *E*, a doorway marks this transition. As indicated by the second step of Figure 1, the interconnection between these areas is represented by a topological map where the nodes mark the areas and the edges the interconnections between these areas. As such, the edges of the topological map represent the navigation affordances, as they indicate the routing alternatives induced by the environment. Mapping these affordances to a set of hypotheses is based on the human detection only by considering the alternatives within the area the human is present in. As a result, these form the first set of hypotheses as listed in Section 3.1. The topological map forms the course discretization of the environment. To make a proper distinction between the various alternatives, areas are not allowed to overlap. In the example scenario presented here, semantic goals refer to neighboring areas of the crossing. In general, when the task of the robot requires it, the topological map can be extended to consider a larger spatial horizon by taking the consecutive areas into account.

For the second set of hypotheses as listed Table 1, the alternatives in the direct neighborhood of the robot are considered. This is indicated by the third step of Figure 1. These alternatives are addressed, adding subareas left and right to the area required for executing the plan of the robot. An example is shown in Figure 2b. Supposing a robot’s navigational task of moving towards area *C*, the area which the robot requires is drawn in the corresponding direction. The desired area is configurable and depends on the movement that is desired by the robot, the velocity of the robot and the expected velocity of the persons within the environment. As the robot might be an objective for a person, a node $B_{f}$ is placed within the area in front of the robot and forms the (possible) collision hypothesis. Now, the alternative routing options consider the passing actions of a person at the left and right side of the robot. As such, areas and their corresponding nodes $B_{l}$ and $B_{r}$ are created at each side of the robot. The remaining areas consider $B_{f}^{\prime }$ and $B_{b}$ at the front and back, respectively. The edges of the graph represent the routing alternatives. Like the static situation, the edges indicate possible hypotheses and show that a route to the back of the robot always directs via the sides of the robot. This is indicated by the fourth step of Figure 1.

### 3.3. Evaluation of the Hypotheses

The final step as indicated in Figure 1 is to quantify how likely the various hypotheses are. Whereas the position estimate of the person of interest is applied to determine the hypotheses set, the progression of a person towards the area corresponding to a hypothesis is chosen as a measure to validate the hypotheses. To determine the progression, the direction component of the estimated human’s velocity vector ${\vec{v}}_{p}$ is compared to the expected direction of movement corresponding to each hypothesis. We opted for a maximum likelihood approach for estimating the likelihood of each hypothesis. We consider prior knowledge about direction preferences equal in all directions. By applying the law of total probability, the ratios between the likelihood of the hypotheses are normalized and as such represented as probabilities. The likelihood $p_{k}\left({\vec{v}}_{H}|H_{k}\right)$ of a velocity vector of a human ${\vec{v}}_{H}$ given the hypothesis $H_{k}$ corresponding to walking pattern *k* measures the progression towards the goal area by comparing the alignment of the human velocity vector ${\vec{v}}_{H}$ and the progression vector ${\vec{v}}_{F_{k}}$ indicating the expected movement direction by

(1) $$p_{k}\left({\vec{v}}_{H}|H_{k}\right)=\max\left(0,\frac{{\vec{v}}_{H}\cdot {\vec{v}}_{F_{k}}}{\left|{\vec{v}}_{H}||{\vec{v}}_{F_{k}}\right|}\right)f_{c}.$$

In this equation, the constraint function $f_{c}$ considers if the person remains in the given field for a certain horizon $t_{f}$ by projecting the person velocity in the direction perpendicular to the expected movement. In the situation of Figure 2a, it is, for example, unlikely that a person who moves from area *B* towards area *D* has a significant velocity component towards area *A* close to the transition between area *A* and *C*. The projection of the person velocity in the direction perpendicular to the expected movement is indicated with ${\vec{v}}_{H⊥}$ and applied to obtain $f_{c}$ according to

(2) $$f_{c}=\left\{\begin{array}{cc} 1 & \mathrm{if}\;v_{pi⊥}<\frac{d_{\mathrm{ef}}}{t_{f}}-\alpha \\ 0 & \mathrm{if}\;v_{pi⊥}>\frac{d_{\mathrm{ef}}}{t_{f}}\\ \frac{-1(v_{pi⊥}-\frac{d_{\mathrm{ef}}}{t_{f}})}{\alpha } & \mathrm{else}. \end{array}\right.$$

Here, α represents a velocity safety margin between instant transition from $f_{c}=0$ to $f_{c}=1$ at $v_{pi⊥}=\frac{d_{\mathrm{ef}}}{t_{f}}$. The distance towards the edge of the field is represented by $d_{\mathrm{ef}}$. For the progression vector as applied in (1), various choices can be made. Given a position, the direct vector to the target could be considered. Within this work, however, it is assumed that the most probable trajectory in which a person moves is a smooth one. As such, streamlines are created which mark the expected trajectory. The direction of these lines indicate the expected movement direction. Normalizing the direction vector gives $|{\vec{v}}_{F}|=1$ and reduces (1) to

(3) $$p_{k}\left({\vec{v}}_{H}|H_{k}\right)=\max\left(0,\cos\left(\theta \right)\right)f_{c}$$

In here, θ indicates the angle between ${\vec{v}}_{H}$ and ${\vec{v}}_{F_{k}}$. The max-function states that there has to be a positive progression towards the goal area to obtain a likelihood greater than zero.

For the validation of the standstill hypothesis $H_{ss}$ as described in Section 3.1, the magnitude of the human velocity vector $|{\vec{v}}_{H}|$ representing the speed is considered. A threshold velocity $v_{min}$ makes a distinction between standing still and walking according to

(4) $$p_{ss}\left({\vec{v}}_{H}|H_{ss}\right)=\left\{\begin{array}{cc} 0 & \mathrm{if}\;|{\vec{v}}_{H}|>v_{\min}\\ 1-\frac{|{\vec{v}}_{p}|}{v_{\min}} & \mathrm{else}. \end{array}\right.$$

The undefined goal hypothesis $H_{\mathrm{UG}}$ represents the completeness of the model. This hypothesis should have a high probability if the other hypotheses are low in likelihood and thus there is no modeled situation that is likely. Therefore, the proof $p({\vec{v}}_{H}|H_{\mathrm{UG}})$ is chosen independent of ${\vec{v}}_{H}$ and is thus a constant value. If there are more likely hypotheses, the UG-hypothesis lowers in probability due to normalization.

## 4. Experiments and Discussion

**Figure 3:**
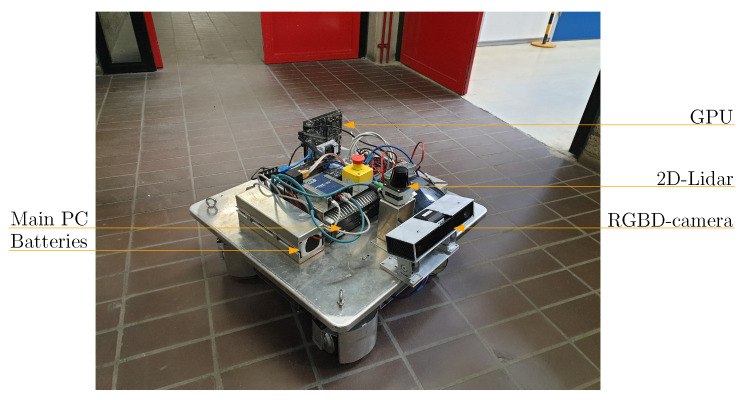
Overview of the prototype Ropod-platform as applied in this work in its target environment. Some of its relevant components are indicated.

To validate the proposed method, multiple experiments were performed at a hallway. The robot utilized in the experiments is a prototype of the ROPOD-platform [2], of which an image is shown in Figure 3. The intention estimation algorithms are executed on an iBase AMI220AF-4L-7700 PC running Ubuntu 16.04. During these experiments, human detections were obtained by providing the OpenPose human detection algorithm [29] with RGB-images of a Kinect v2 RGBD-camera. The detection algorithm ran on a Jetson TX2 board, with a detection rate of 3–4 [Hz]. By utilizing the depth-channel of the camera, the observed position of the person was determined as the average distance with respect to the camera of the human body joints as obtained by the OpenPose detector. To compensate for measuring the front of the human joints instead of their center, the typical human body radius is added to the average distance. This radius is assumed to be 0.15 [m]. To obtain the position and velocity estimation of the human, the detections were fed to a constant velocity Kalman filter containing a white noise acceleration model. For details on the configuration of the Kalman filters used in the experiments, the reader is referred to, e.g., [28,30]. ROS [31] was chosen as middleware, and the ROS-AMCL package [32] provided the robot’s localization. The implementation and videos of the experiments are available in the public code repository accompanying this paper (https://github.com/tue-robotics/human_intention_prediction (accessed on 8 June 2021)). The configuration of the model variables adopted during the experiments is reported in Table 2. In this table, the reserved area around the robot yields a safety margin around the setup and can be adapted based on the preferred motion direction by the motion planner. The search and consideration area, the side margins of the field and the time constraint of remaining inside a field consider a prediction horizon of a couple of seconds and a typical human walking speed of 1.4 [m/s] [33]. The UG-likelihood is chosen low in comparison with the likelihood of the other hypotheses because it is assumed that human navigation goals relate most of the times to the semantic areas identified in the map.

In total, three sets of experiments are performed in real-time. In the first set of experiments, the performance of the model is analyzed on a single crossing. A detailed explanation is given for a few experiments, while the robustness is demonstrated by repeating the experiment with various persons moving on a crossing. In the second set of experiments, the scalability is demonstrated by adding an extra point of interest, which is detected during runtime. Whereas in these experiments, for demonstration reasons the robot was not moving, in the last set of experiments, the functionality is demonstrated with a moving robot.

### 4.1. Experiment 1: Single Crossing

**Figure 4:**
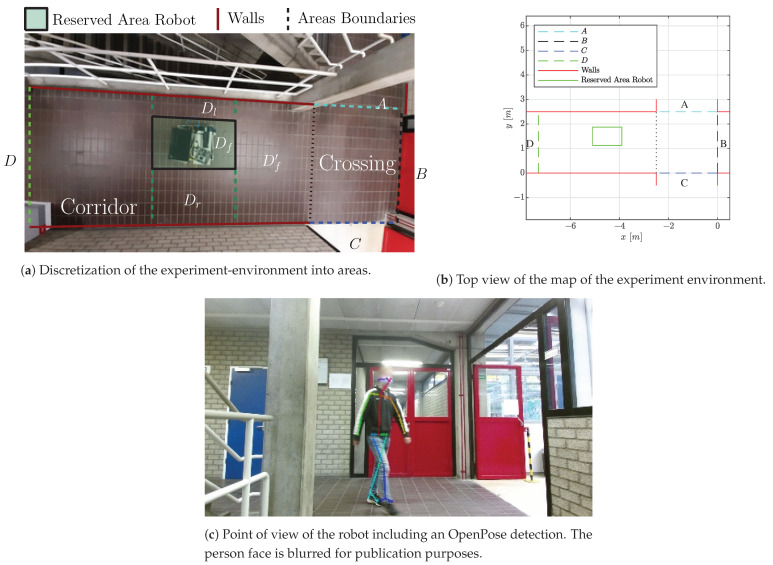
Visualization of the experiment-environment. The detection originates from [29].

In the first experiment, the intention estimation algorithm is demonstrated and analyzed on a single crossing, which the robot is supposed to cross. A few cases are demonstrated in detail, followed by a set of tests with various persons. The hallway considered in this experiment is visualized in Figure 4. A coarse discretization of the hallway is performed to distinguish different navigational goals for a human as displayed in Figure 4a,b. Five possible navigational goals are identified: the robot itself, door *B*, corridors *A* and *C*, and corridor *D*, which is reachable via the left side $D_{l}$ or right side $D_{r}$ of the robot area $D_{f}$. The dashed lines indicate the boundaries of the areas.

#### 4.1.1. Experiment 1.1: Human Passing a Robot

**Figure 5:**
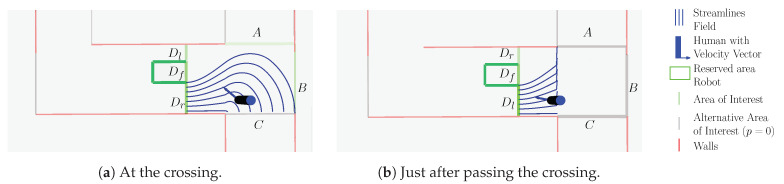
Visualization of the hypothesis where a person coming from corridor C takes a left turn at the crossing to move via the right side of the robot $D_{r}$ towards corridor *D*. The brightness of the area of interest, indicated in green, correlates to the probability of moving towards this area. A brighter color indicates a higher probability.

The experiment consists of validating the hypothesis that a human walks from corridor *C*, taking a left on the crossing, passing the robot on the left side $D_{l}$ towards corridor *D*. The visualization of this hypothesis is shown in Figure 5 at two different moments. In the figure, the walls, the robot reserved area and both the position and velocity estimate of the person are indicated. Green lines indicate the different hypotheses. A brighter green line indicates a higher certainty associated to that hypothesis. Streamlines, indicating the expected movement-directions according to the hypothesis, are visualized with blue lines and constrained by their area boundaries by means of the walls, the robot and the transition between areas. For visualization purposes, the streamlines are determined for the entire area, but in practice, given a position estimate of a person, the expected movement is determined at the specific position only. The direction of the streamlines is compared to the observed direction to quantify the progression according to (3). The figure shows that the model predicts, with a significant probability, that the person will pass the robot on its right side as the green line corresponding to area $D_{r}$ is significantly more bright. As there is still a reasonable probability of the person moving towards the robot when considering a navigational task of the robot, this risk can be reduced by the robot by moving towards the wall opposite to the person.

#### 4.1.2. Experiment 1.2: Collision Course

**Figure 6:**
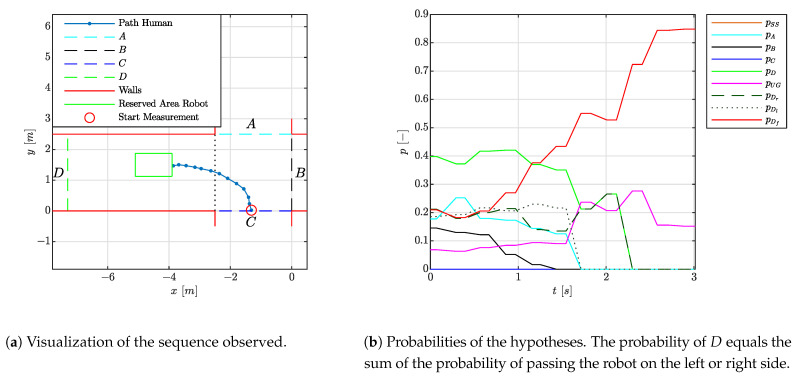
Visualization of the experiment where a person is walking towards the robot.

Figure 6 reports on the case in which a person moves towards the robot. The left side of the figure indicates the division of the environment into areas, the reserved space of the robot and the estimated trajectory of the person. The hypotheses set consist of the movements to areas *A*, *B*, *C* and *D*, a standstill and Undefined Goal. When the person moves towards the robot, refined hypotheses as to whether the person is passing the robot on the left, right or a collision are also reported. In this situation, the probability of a person moving towards area *D* is formed by the routes which pass the robot on the left or right side, hence the probability of *D* equals the sum of the probability of passing the robot on the left and the right side. On the right side of the figure, the progression of the probability *p* of the hypotheses over time *t* is shown. Note that the route to *D* consists out of two alternatives, namely passing the robot via either the left or the right side. Hence, the probability of a person going into direction *D* consists out of the sum of the probabilities of these two alternatives. It is observed that after 0.5 [s] the collision hypothesis is among the most dominant ones and after about 1.5 [s] it is the dominant one. Given a robot which moves at average human walking speed of 1.38 [m/s] [33] and considering a robot deceleration of 1.5 [m/s${}^{2}$], this is well in time to bring the robot to a standstill. As the collision hypothesis is among the dominant ones within the first second, the robot is expected to take this risk into consideration during its navigation task even before the first second. To gain more time, the robot shall lower its velocity when facing this situation.

#### 4.1.3. Experiment 1.3: Standstill

**Figure 7:**
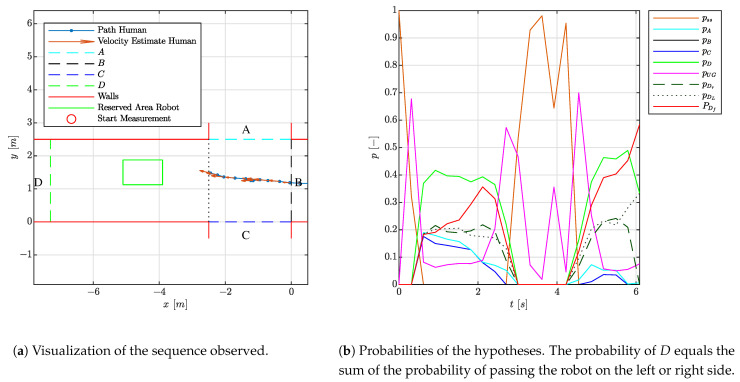
Visualization of the experiment where a person enters the crossing, waits for a moment and continues its route in forward direction.

The third set of hypotheses, as shown in Table 1, indicates that a person might be standing still. This is the case, for example, when the person is doubting about the route to take. Therefore, this experiment validates the standstill hypothesis. In this experiment, a person moves towards the crossing, stands still for a bit and then continues their route. The results are shown in Figure 7. The average computation time to determine the hypothesis equals $6.8\times 10^{-2}$ [s]. As can be observed in Figure 7a by the higher density of the human position indication, the person was standing still more or less at the middle of the crossing. In the corresponding period, as indicated in Figure 7b, the standstill hypothesis is dominant. At the transition from walking to a standstill and vice versa, the UG-hypothesis is dominant for a moment. As a result, some conservatism is required by the robot in these transition periods as no clear indicators for a specific movement are found. Afterwards the correct hypothesis of a movement towards the robot becomes dominant.

#### 4.1.4. Experiment 1.4: Indecisive Person

**Figure 8:**
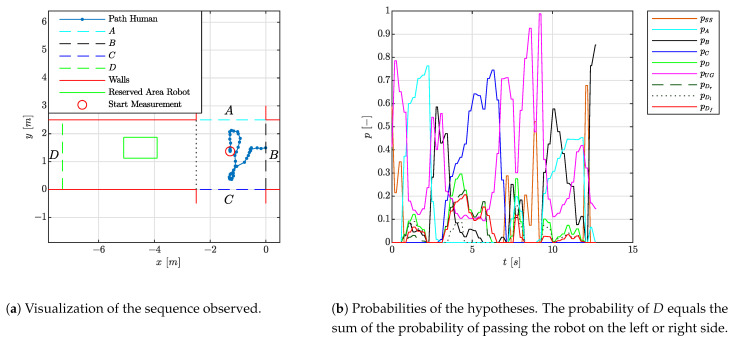
Visualization of the experiment where a person is searching for its route.

The fourth experiment shows an indecisive person as this person is not instantly choosing one area over another. This behavior is confirmed in Figure 8 as the UG-hypothesis is dominant. With such a prediction, caution in the robot navigation is required. In the intermediate phases, the probabilities of going to either corridor *A*, *C* or *B* increase as the person initiates a movement in those directions. Due to this movement, the UG hypothesis decreases. In the transitions, in correspondence with the previous experiment, a settling time of approximately 1 [s] is observed. For a robot with a navigational task, this is very useful, as this requests caution when necessary and permits proper progression in the task where possible.

#### 4.1.5. Experiment 1.5: Various Persons

To demonstrate the robustness and confirm the settling time of the proposed method, the experiment is repeated with 9 separate persons which were asked to move over the crossing and randomly choosing a direction. The results are provided in Table 3. In this table, the routes are indicated by their origin and their destination, the frequency of the execution of the movement, the average settling time $t_{s}$ of the correct hypothesis between the first detection and the moment the correct hypothesis is considered as the most likely one. Further, the percentage of correct estimations is provided.

**Figure 9:**
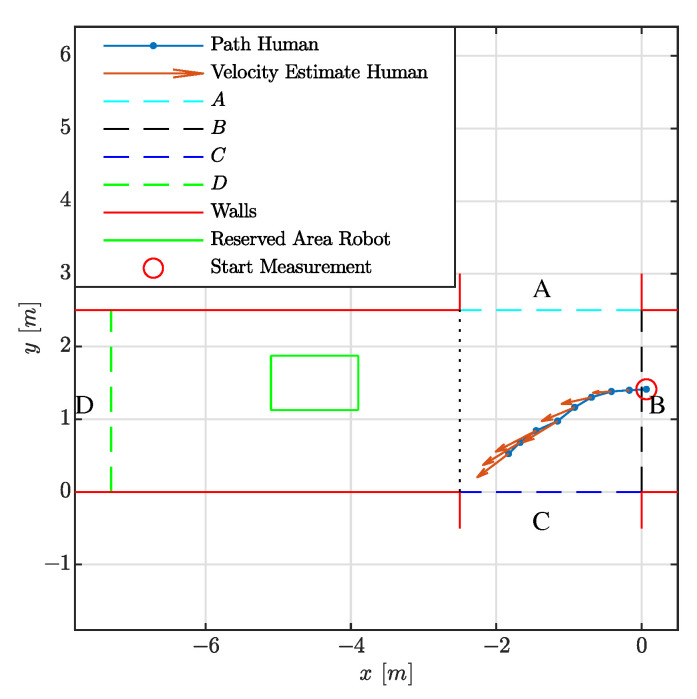
Situation of Table 3 where a person moves from area *B* to area *C* and the hypothesis is not correctly validated.

In total, 35 movements over the crossing were registered with an average settling time of 0.9 [s]. This is in correspondence with the findings in the previous experiments and given the reasoning of Section 4.1.2 considered well in time for the robot to make proper decisions about which route to take. Two out of three times, difficulties were seen for persons moving from area *B* to *C*. Therefore, an experiment in which an incorrect estimation of the human’s intention was made is visualized in Figure 9. The figure includes the velocity estimation of the person. Here, it is seen that a significant component of the velocity estimate is directed towards area *D* along the trajectory of the person, and as such the hypotheses towards area *D* are more likely. Improvements to correctly validate the corresponding hypothesis are sought in finding out the actual walking patterns of persons, as apparently persons tend to move more to the right side of the corridor of area *C*. These improvements are left for future work.

### 4.2. Experiment 2: Online Adaptation

**Figure 10:**
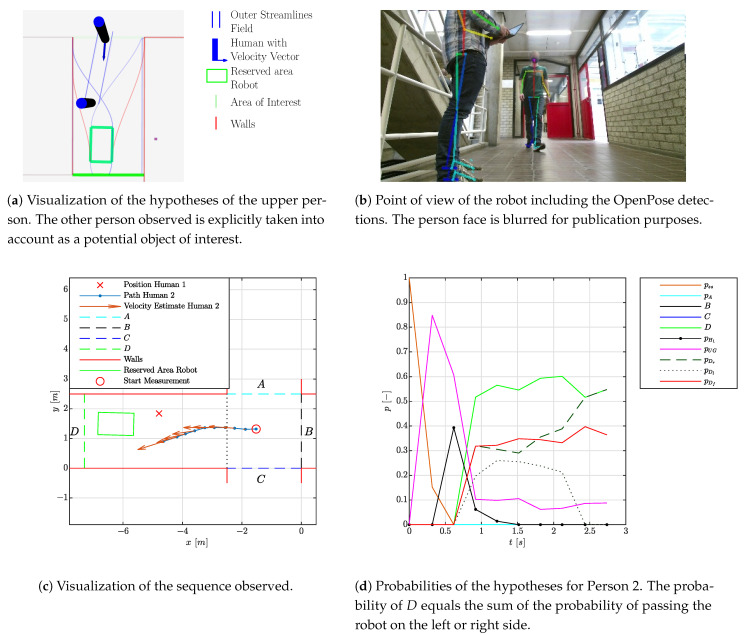
Visualization of the experiment with an a priori unknown object. The detections originate from [29].

Though a semantic map is assumed to be known a priori, other objects such as a person or an unmodeled coffee machine could be present and considered as potential objects of interest. As a result, these objects need to be considered as alternative hypotheses. To demonstrate the scalability of our approach, compared to the first set of experiments, an extra person is added to the situation. Whereas the first person $P_{1}$ in the situation is standing still, the second person passes the first person and the robot. The situation is visualized in Figure 10b,c. The corresponding hypotheses for the second person are given in Figure 10a,d. For the second person, these figures indicate that the first person could be an object of interest. The corresponding hypothesis is indicated by a straight line towards the other person. Halfway through the sequence, indicated by the velocity vector, the probability correctly drops as the first person is (about to be) passed. At the same moment, the movement to the right of the robot side is correctly validated.

### 4.3. Experiment 3: Dynamic Situation

**Figure 11:**
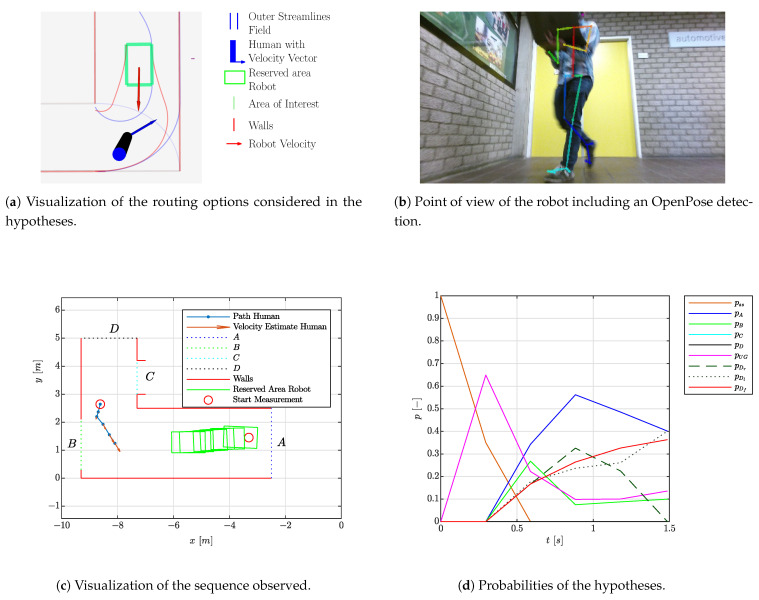
Visualization of the experiment with a moving robot and a moving person. The probability of A equals the sum of the probability of passing the robot on the left or right side. The detection originates from [29].

The final experiment shows a situation where the robot is navigating through the environment while hypothesizing about the intentions of the person observed. During the experiments, AMCL [32] was applied to determine the localization and the “tube navigation algorithm” of [34] provided the navigation. Both could be replaced with other localization or navigation algorithms. An overview is shown in Figure 11. The environment and the trajectory applied by both the robot and person are shown in Figure 11c. The crossings behind *A* and *C* as well as the door behind *B* and stairs behind *C* determine the discretization of the map. As the robot needs to be passed by the human towards area *A*, three alternatives are foreseen: the robot itself could be a target, or alternatively the robot could be passed on the left or right. As a result, the latter two passing alternatives lead to area *A*. The robot was moving towards area *D*, while the person was moving towards area *A* via the right side. A moment where the hypothesized routing alternatives are visualized can be observed in Figure 11a.

The evolution of the probabilities in Figure 11d indicate that the probabilities of the hypothesis where the robot is passed on the right as well as the probability of the hypothesis where the robot is the target of the person have high probabilities. This is expected because significant parts of both routing alternatives are similar. Future research could take these alternatives into account in the navigation of the robot: for the case where the person is passing the robot, by moving the robot to the side opposite to the side where the person is passing the risk of occlusions will be reduced. Further, more space is given to the person passing by, probably making the robot movement more intuitive to the person. Now, as could be observed in Figure 11b, the space for the person to pass is relatively tight. For the alternative case where the robot is considered as a goal of the person, the system should slow down and eventually come to a standstill to prevent collisions.

## 5. Conclusions & Recommendations

In order to make predictions about human walking intentions in the context of robot navigation, this work has proposed a model that explicitly addresses expected human movements as imposed by an indoor environment. Rather than considering the geometric accuracy, it is shown that the model expresses an explicit relationship between measurements and map elements to provide predictions on a semantic level. As a result, an explainable model is obtained. By showing how the proposed approach performs in different scenarios and in real-time (including a moving robot and the online hypotheses generation based on unmodeled objects observed at runtime), the scalability and applicability to various configurations of the environment is demonstrated. The robustness was shown with a set of static experiments: 33 out of 35 experiments demonstrated that the plausible routing alternatives are correctly taken into consideration, and in case of a likely collision, the right conclusion was drawn well in time to bring the robot to a standstill.

Since the various possible routing directions of a human are related to the environmental map, we argue that the results of our model can be easily integrated in navigation algorithms. The actual integration of these methods into navigational contexts is considered as a relevant topic for future work. By having an higher update rate of the person detection and tracking, faster person movements can be taken into account and a more accurate estimate of the (orientation of the) person’s motion could be used as an indicator of their intentions. Attention should be paid to changes of behavior caused by robot–person and person–person interactions, as it is expected that the motion patterns of persons change due to these interactions. Furthermore, the cases in the experimental validation where incorrect conclusions were drawn indicated some situations where people tend to walk towards the right-hand side of the consecutive corridor. This type of behavior should be included during future work as well.

## Figures and Tables

**Table 1:** Hypotheses set considered.

Hypotheses	Notation	Alternatives Considered
1	$H_{k}$	All turns *k* on a crossing
2a	$H_{k_{\left\{l,r\right\}}}$	Passing the robot on the left or right side
2b	$H_{f}$	Robot is a goal
3	$H_{ss}$	Standstill
4	$H_{UG}$	Other goal

**Table 2:** Settings applied within the algorithm.

Setting	Value
(length, width) of reserved area robot	(1.2,0.75) [m]
search area around (robot, human)	(3.0,4.0) [m]
likelihood $H_{UG}$	0.3 [-]
$v_{min}$	0.1 [ms${}^{-1}$]
α	0.4 [m/s]
$t_{f}$	1.0 [s]

**Table 3:** Experimental results when testing on a single crossing as visualized in Figure 6 and Figure 8.

Origin	Destination	Frequency	% Correct	$t_{s}$
*A*	*B*	1	100	0.3
*A*	*C*	3	100	0.70667
*A*	$D_{r}$	6	100	0.87667
*A*	$D_{f}$	1	100	2.4
*B*	*A*	1	100	1.48
*B*	*C*	3	33.3	0.6
*B*	$D_{r}$	4	100	0.75
*C*	*A*	7	100	0.64714
*C*	*B*	2	100	0.61
*D*	*A*	1	100	0.59
*D*	*B*	3	100	1.3033
*D*	*C*	3	100	1.6067

## Data Availability

Not applicable.

## Abbreviations

The following abbreviations are used in this manuscript:

AGV	Automated Guided Vehicle
UG	Undefined Goal
